# Supplementary material for: Implementation fidelity of school oral health programs at a District in South Africa
Source: PLoS One. 2020 Nov 17;15(11):e0241988. doi: 10.1371/journal.pone.0241988 (PMC7671500; doi:10.1371/journal.pone.0241988)
Supplement: S1 Appendix — (DOCX) [file pone.0241988.s001.docx]

# S1 Appendix. Presentation of fidelity elements and how they were measured.

| **Fidelity Elements** | **No schools adhering** | **Definition** | **Measurement Classification** |
| --- | --- | --- | --- |
| **Content**  Community Assessment  Resource Assessment  Screening  Brushing program  F/S children 7/8 yrs  F/S children 12/13 yrs  ART  Dietary Health Practice  Oral Health training for staff  Collective implementation | 0  0  2  5  6  7  2  2  3  1 | Key activities of the program.  Schools scoring 5 ≤ on content =1Adherence  Schools scoring < 5 on content =0 Adherence | **ADHERENCE** |
| **Coverage** | 7 | Number of pupils reached / Total number of children in grades 1 & 7 per school.  (More than 50% school coverage = 1; less =0) |  |
| **Duration** | 8 | Number of hours spent at a school per day.  (Minimum 4 hours = 1; less =0) |  |
| **Frequency** | 5 | Number of times a school was revisited in a year.  (Minimum 2/year = 1; less =0) |  |
| **Quality**  Preparedness  Interaction with teams  Communication | 7  8  7 | Quality of delivery  (Yes = 1; No= 0) | **POTENTIAL MODERATORS** |
| **Facilitation Strategy**  School staff support  Dental assistance availability  Functioning equipment  Availability of water | 8  0  3  8 | Facilitation strategies  (Yes =1; No = 0) |  |
| **Participant responsiveness**  Stakeholder engagement  Stakeholder satisfaction  Perception of relevance  Parental participation | 5  3  9  4 | Participant responsiveness  (Yes = 1; No = 0) |  |
