# Supplementary material for: Implementation fidelity of school oral health programs at a District in South Africa
Source: PLoS One. 2020 Nov 17;15(11):e0241988. doi: 10.1371/journal.pone.0241988 (PMC7671500; doi:10.1371/journal.pone.0241988)
Supplement: Attachment — Submitted filename: Response to Reviewers.docx [file pone.0241988.s003.docx]

**Dear Reviewers**

**Re article:** **Implementation fidelity of School Oral Health Programs at a District in South Africa.**

Thank you very much for your review of our manuscript and for the comments which aim to improve its clarity and flow. We have considered all the comments and have included below a point-by-point response to each of them, for each reviewer. We have also made the necessary changes to the manuscript on the pages and line numbers indicated.

**Reviewer comments and responses**

Reviewer #1: The article is well written.
#1 - The study measures fidelity of implementation. However, it is never made clear exactly what is being implemented. What are the components of the school dental health program? This information could be provided in the Background section, or in the Methods section, or perhaps in a table.

**Addressed as recommended in : Page 5; lines 111-114 & Page 7; Appendix 1**

1. Who are the dental hygienists? What is their educational background, and what training do they receive to implement the school dental health program?

**Addressed as recommended in : Page 4; lines 100-104.**

2. How many schools do they visit in a day or in a week? How long are they in each school?

**Addressed as recommended in : Page 5; lines 109-117.**

3. How many students do they need to see during a visit to a school?

**Addressed as recommended in : Page 5; lines 109-117.**

4. What are all the activities that are to carry out during a visit to a school? Do their activities include promotion / education about dental hygiene? Do they talk to groups or classes of children about dental hygiene?

**Addressed as recommended in : Page 5; lines 109-117.**

5. What is the role, if any, of school staff such as teachers and principals in implementation of the school dental health program?

**Addressed as recommended in : Page 5; lines 109-117.**

#2 - What Department or Ministry in the Government is responsible for the program? Do they carry out any routine monitoring of implementation of the program?

**The Gauteng Department of Health is responsible for the program, comments addressed in: Page 4; lines 102-103 & Page 6; 148-149**

#3 - The conclusions / recommendations could be strengthened. How can implementation of the program be improved?

**Conclusions and recommendations have been addressed in: Page 17; lines 375 -389.**

**Thanking You**

**M. Molete**
